# The Impact of Animal Models and Strain Standardization on the Evaluation of Tuberculosis Vaccine Efficacy

**DOI:** 10.3390/vaccines13070669

**Published:** 2025-06-21

**Authors:** Jiazheng Wei, Junli Li, Xiaochi Li, Weixin Du, Cheng Su, Xiaobing Sheng, Yang Huang, Jinsong Wang, Qun Niu, Guoqing Chen, Wei Tian, Aihua Zhao, Miao Xu

**Affiliations:** 1 College of Life Sciences and Biopharmaceuticals, Shenyang Pharmaceutical University, Shenyang 117004, China; weijiazheng0604@163.com; 2 Division of Tuberculosis Vaccine and Allergen Products, Institute of Biological Product Control, National Institutes for Food and Drug Control, Beijing 102629, China; lijunli@nifdc.org.cn (J.L.); lixiaochi0928@163.com (X.L.); duweixin@nifdc.org.cn (W.D.); jhzz@163.com (C.S.); shengt99@163.com (X.S.); 3 State Key Laboratory of Drug Regulatory Science, National Institutes for Food and Drug Control, Beijing 102629, China; huangyang@nifdc.org.cn (Y.H.); wangjinsong@nifdc.org.cn (J.W.); niuqun158@126.com (Q.N.); chengq@nifdc.org.cn (G.C.); 4 Key Laboratory for Quality Research and Evaluation of Biological Products, National Medical Products Administration (NMPA), Beijing 102629, China; 5 Key Laboratory of Research on Quality and Standardization of Biotech Products, National Health Commission (NHC), Beijing 102629, China

**Keywords:** tuberculosis, tuberculosis vaccine, *Mycobacterium tuberculosis*, animal models, standardization research

## Abstract

Tuberculosis (TB) remains one of the most significant challenges to global public health. Vaccine development is a critical strategy for the prevention and control of TB. However, evaluating the protective efficacy of TB vaccines faces numerous challenges, particularly in the selection of animal models and bacterial strains. Variations in animal models, challenge strains, challenge routes, and doses can significantly impact the outcomes of preclinical evaluations. This article highlights the importance of standardizing preclinical evaluation models, summarizes the animal models and challenge strains used in novel TB vaccine candidates, efficacy studies, and discusses the advantages and limitations of commonly used animal models in TB vaccine research. It also points out the differential performance of various animal models in simulating protection and pathology. Given the current limitations of using a narrow range of challenge strains and the lack of standardized infection routes and doses, this article calls for the establishment of more standardized challenge strains and the development of standardized evaluation models to improve the reliability and generalizability of new TB vaccine efficacy assessments.

## 1. Introduction

TB is a chronic infectious disease that severely threatens human health. According to the 2024 WHO report, TB has become the leading infectious cause of death globally, with 8.2 million new cases reported in 2024 [1]. These alarming statistics underscore the urgent need for more effective and immediate measures to combat TB. Although the *Bacille Calmette-Guérin* (BCG) vaccine, introduced in 1921, has been the primary tool for TB prevention, its protective efficacy varies significantly across populations and is limited in preventing pulmonary TB in adults. Therefore, the development of new TB vaccines has become an urgent global public health priority.

As a key strategy for TB prevention and control, vaccine development continues to face significant challenges, particularly in the standardization of animal models and challenge strains during the preclinical phase. Currently, the use of a limited range of challenge strains fails to account for the complex geographical and genetic diversity of MTB, which impacts the broad applicability of vaccines. The lack of diverse, standardized strains and the significant differences in protective efficacy observed across animal models, coupled with the absence of unified evaluation criteria, limit the comparability and reliability of research findings. In the past decades, numerous animal models have been developed, and various pharmacodynamic evaluation systems were used for TB vaccines. We aim to review the application and development of animal models commonly used in TB vaccine efficacy evaluations, explore the diversity and standardization of challenge strains, analyze the scientific gaps in animal models and strain standardization, propose a multidimensional evaluation framework, and provide insights into future directions for TB vaccine development. By addressing these issues, this article aims to provide a theoretical foundation and technical support to accelerate the development of novel TB vaccines.

## 2. Evaluation Models for TB Vaccine Protective Efficacy

### 2.1. Commonly Used Models for Protective Efficacy Evaluation

Currently, the preclinical studies of TB vaccines in clinical trials exhibit significant differences, including the animal models, challenge strains, challenge routes, and doses. The most commonly used animal models are C57BL/6 mice, BALB/c mice, guinea pigs, and rhesus macaques, which are widely employed in the evaluation of TB vaccine protective efficacy.

However, the construction methods and evaluation approaches for these models vary, and there is currently no standardized model for researchers to reference. The challenge strains, infection routes, and doses may differ even within the same animal model (Table 1). Notably, the standard strains H37Rv and Erdman are widely used in TB vaccine evaluation models and have received significant attention, while other prevalent strains are less commonly used, and the adaptability of clinically isolated strains is largely overlooked [2]. Given the inherent complexity of *Mycobacterium tuberculosis* (MTB), the intricate interactions with the host, and the unclear immune mechanisms, the lack of immunological markers directly correlated with protection means that evaluations based solely on standard or sensitive strains are insufficient. The failure of the MVA85A vaccine in a Phase IIb clinical trial in South Africa, despite its efficacy in mice and guinea pigs, underscores the necessity of comprehensive preclinical pharmacodynamic evaluation [3,4]. There was significant analysis of the preclinical models that suggested the vaccine may not have worked in humans [5]. Owing to the complex interplay between MTB and host immunity, conventional animal models often fail to recapitulate the heterogeneity of tuberculosis. To address this limitation, Collaborative Cross (CC) and Diversity Outbred (DO) mice have been developed for this research domain. The CC mice constitute a reproducible cohort of genetically diversified mice exhibiting broad phenotypic responses to infection. Consequently, CC mice can be used to determine the correlates of protection and establish vaccine strategies to protect a larger proportion of genetically diverse individuals, rather than optimizing protection for a single genotype [6]. Because immune and bacterial traits may be associated with genetic variations in the mouse genome, CC mice become a unique population for identifying specific host-pathogen genetic interactions that influence pathogenesis, thereby enabling more accurate detection of vaccine protective efficacy. Currently, a platform has been established to link bacterial genetic requirements with host genetics and immunity, confirming the polygenic control relationship between CC mice and TB and identifying genetic loci that govern different aspects of pathogenesis [7,8]. DO mice are generally formed by random heterogeneous mating of mice from eight highly diverse original strains and are widely used in research such as clinical trials, transcriptomics, and proteomics [9,10,11]. Studies have found that candidate genes in DO mice may be the main regulators of complex host-pathogen interactions leading to granulomatous necrosis and acute inflammation in tuberculosis [12]. Therefore, DO mice have great potential in the evaluation of vaccine protective efficacy. Future studies may integrate single-cell omics with spatial transcriptomics to further dissect host-pathogen interactions and elucidate mechanisms underlying latent infection and disease pathogenesis [13,14,15].

In conclusion, establishing standardized challenge strains and animal models for the efficacy evaluation of diagnostic, preventive, and therapeutic products is crucial for obtaining reliable and accurate evaluation data, thereby accelerating the clinical translation of innovative TB products.

Mice and guinea pigs are widely used in the development of vaccine candidates. Mice are generally employed for antigen screening and preliminary evaluation of vaccine efficacy, while guinea pig models are often used for further efficacy assessment of vaccine candidates that have passed initial screening in mouse models. Common animal infection models include active TB models and latent TB infection (LTBI) models. The active TB model is most frequently established using inbred mice with a C57BL/6 background. After acute infection, MTB proliferates actively in these mice, exhibiting strong virulence and high infectivity. In preclinical studies, active TB models using mice and guinea pigs can only simulate one or a few characteristics of human TB, such as pathological changes, bacterial load in organs, disease progression, blood changes, or immunological parameters. LTBI models are often used to evaluate the ability of TB vaccines to reduce the activation rate of LTBI, predict their long-term impact on the incidence of active TB, simulate the mechanisms by which latent infections progress to active TB, and assess the blocking effects of new vaccines (e.g., M72/AS01E [52] and AEC/BC02 [17]) on the activation of LTBI. Due to the lack of standardized LTBI animal models for research, the mechanisms of MTB latent infection and reactivation remain unclear. Additionally, to align scientific research more closely with the principles of replacement, reduction, and refinement (3R) in animal welfare ethics, low-sentience animal models such as fruit flies and zebrafish can also be used for TB vaccine evaluation and mechanistic studies [53,54,55].

In the evaluation of TB vaccine efficacy, in addition to commonly used strains such as H37Rv and Erdman, other screened strains with strong virulence and stable states (e.g., CMCC 95052 [16,17]) are also included. Different strains exhibit varying affinities for the same organs, leading to differences in pathological manifestations across organs, which is one of the significant challenges in standardizing evaluation models. BCG, an attenuated live vaccine strain of *M. bovis*, lacks key virulence genes and cannot form granulomas or infectious pathological features in the host. The H37Ra, with defective expression of related virulence genes, cannot simulate the pathological damage of TB infection, making it unsuitable for evaluating the true protective efficacy of vaccine candidates. Therefore, evaluating vaccine protection using a single challenge strain has limitations.

Different challenge routes can also lead to variations in organ pathology. In theory, animals infected via aerosol, intranasal, or intratracheal routes often exhibit more severe lung lesions, while those infected via subcutaneous or intraperitoneal routes tend to show more severe spleen lesions. Aerosol infection can better simulate the real environment of human respiratory MTB infection, and the infection dose can be adjusted by controlling the bacterial concentration, airflow speed, and mouse exposure time in the nebulizer chamber. A low-dose subcutaneous infection in guinea pigs, combined with continuous use of anti-TB drugs such as isoniazid-rifampin tablets, can establish an LTBI model [17,56]. This model is often used to study the prevention of latent infection reactivation, the induction of long-lasting immunity, and the immune-enhancing effects of new vaccines. The Cornell model is limited due to its poor reproducibility and significant variability in TB recurrence rates, making the establishment of new, stable LTBI and TB recurrence models a significant challenge.

### 2.2. Animal Models of Active Tuberculosis

**Figure 1:**
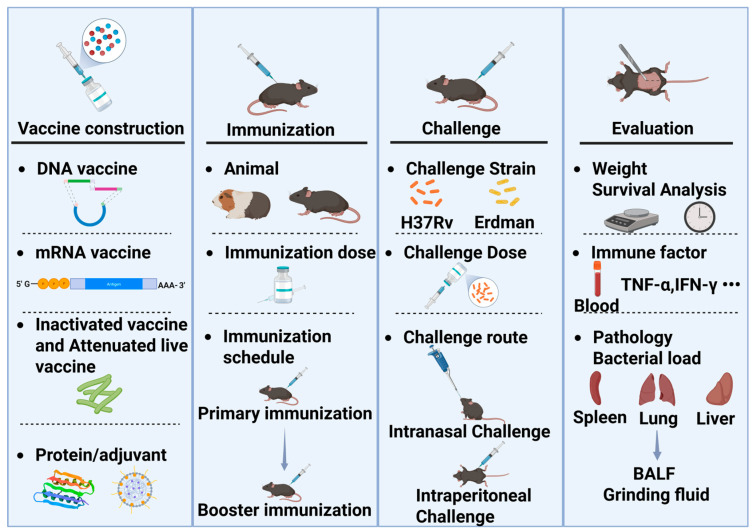
The evaluation process of TB vaccine efficacy in rodent models. BALF: Bronchoalveolar lavage fluid.

The evaluation of TB vaccine efficacy encompasses animal models, challenge strains, and evaluation metrics (Figure 1). The process begins with vaccine development, where the dosage and immunization schedule may vary depending on the vaccine. Typically, after determining the appropriate immunization dosage and schedule through exploratory studies, challenge experiments can be conducted. The dosage and immunization schedule need to be optimized independently, as variations in primary immunization, booster immunization, and timing can lead to differences in outcomes. The first step is to select the appropriate animal species for challenge experiments. Mice and guinea pigs are commonly used for efficacy evaluation. Mice are also utilized to assess the acute toxicity and drug distribution of TB vaccines, while guinea pigs are used to evaluate skin hypersensitivity reactions, such as the Koch’s phenomenon. The source, batch, and age of the animals must be consistent. Once the animals are immunized, they are transferred to a Biosafety Level 3 (P3) laboratory for the challenge experiment.

Many animals have been used to decipher the mechanisms related to TB, among which mice, guinea pigs, and macaques have performed exceptionally well in evaluation models [57]. Mice are commonly used to study the immune or inflammatory mechanisms of TB, but there are significant differences in innate gene expression patterns and adaptive immunity between humans and mice. The disease outcomes observed in mouse models do not fully reflect the symptoms seen in human TB [58,59,60].

Bacterial load and pathological changes are easily monitored and used to assess the impact of TB interventions in guinea pigs infected with MTB. Currently, validating the protective efficacy of vaccines through low-dose aerosol guinea pig models is an essential component of preclinical data for TB vaccines [61]. The pathological lesions formed inside and outside the lungs of MTB-infected guinea pigs have been extensively studied, and guinea pigs can develop granulomas structurally similar to those in humans [62]. Therefore, they are suitable for studying the pathogenesis of TB as well as the evaluation of vaccines and drugs, particularly as challenge models for assessing the efficacy of TB vaccines [63].

Mice are commonly used for screening immunogenicity due to their cost-effectiveness, ease of handling, and availability in TB vaccine development. Guinea pigs or mice should be selected for vaccine efficacy evaluation according to actual conditions. Guinea pigs and mice have their respective advantages in efficacy testing. Mice have a wide variety of strains, providing rich animal models such as susceptible strains, hybrid strains, and outbred strains. Guinea pigs, on the other hand, exhibit pathological characteristics closer to humans and have more advantages in long-term immunity and latent infection models. If a vaccine demonstrates promising protective efficacy, further evaluation can be conducted in guinea pigs or large animal models (such as non-human primates, NHPs) as needed, including assessments of long-term protective efficacy. Although expensive, these models can more closely simulate human immune responses, providing reliable testing of the protective efficacy of potential TB vaccines [64]. Clinical data show that the immune responses induced by MTBVAC immunization in NHPs and humans are consistent, suggesting that the protection provided by the MTBVAC vaccine in macaques will be applicable to humans [65,66,67,68]. Animal models established in rhesus macaques and cynomolgus macaques have been used to evaluate the efficacy of novel TB vaccine candidates, and the outcomes of MTB infection in these models have been extensively studied [69,70,71,72]. Research has found that cynomolgus monkeys exhibit symptoms similar to humans, primarily latent infections, while MTB infection in rhesus macaques spreads rapidly in the lungs, almost always progressing to active TB; this feature allows for differentiated evaluation in vaccine prevention [73,74]. It is noteworthy that the challenge dose for evaluating vaccine efficacy should strike a balance within a reasonable range, simulating natural human MTB infection while retaining the ability to infect animals. However, there is currently no standardized animal evaluation model, and challenge doses lack unified standards, making comparisons between similar studies less reliable.

No single animal model can fully represent all aspects of human TB or replicate all the histopathological features observed in natural human TB [75,76]. However, each animal model has its own characteristics, and different models may yield inconsistent results in TB vaccine efficacy studies [77]. Selecting appropriate and high-quality animal models is crucial for the quality of vaccine efficacy validation. Therefore, establishing standardized animal efficacy evaluation models is necessary to provide technical guidance for vaccine design.

### 2.3. Animal Models of LTBI

LTBI murine models are critical for evaluating TB vaccines and prophylactic drugs, particularly in studying interventions to prevent reactivation [78,79,80]. Currently, LTBI models primarily include the Cornell model and chronic low-bacterial-burden infection models. Low-dose persistent infection in mice partially mimics certain features of human TB. However, these models have significant limitations, including lacking granuloma formation, prolonged establishment times, marked individual variability, and inconsistent TB reactivation rates, making it challenging to elucidate the immunological mechanisms underlying LTBI. Researchers have developed latent infection models by transient genetic inactivation of essential protein-related virulence genes; while these models offer improved stability, they fail to fully replicate LTBI or the persistent infection state following TB chemotherapy [81]. In contrast to anti-TB drug interventions, LTBI mouse models based on immune control mechanisms more closely resemble the burden state of LTBI. However, achieving this requires prior administration of TB prophylactic vaccines to establish sustained immunological containment [82].

Researchers have employed CBA/J, DBA/2, and C3H/HeJ mice to construct susceptible models for predicting disease outcomes via IFN-γ release assays (IGRA), aiding in distinguishing active disease from LTBI and quantifying reactivation risks in LTBI carriers [83]. BALB/c and C3HeB/FeJ have been successfully used to establish mouse models of LTBI, verifying the effectiveness of various novel anti-TB drugs and combination treatment regimens [84].

Guinea pigs have also been utilized to model LTBI, but their inherent MTB susceptibility often leads to rapid progression to active TB, marked by weight loss and mortality. Xu M. et al. [85] established an LTBI model by subcutaneously infecting Hartley guinea pigs with 5 × 10^3^–1 × 10^4^ CFU of MTB (CMCC95052), demonstrating the efficacy of AEC/BC02 and AEC/BC03 vaccines in reducing bacterial burden and mitigating organ pathology. Another study successfully modeled LTBI using 500 CFU H37Rv infection combined with isoniazid (INH) and pyrazinamide (PZA) treatment, linking tuberculin (PPD) reactivity to TB recurrence risks [86]. Recent advancements in guinea pig models have enhanced disease assessment and immunopathological studies.

NHP LTBI models recapitulate the natural progression of human LTBI infection, exhibiting clinical features closely aligned with human LTBI manifestations [87]. CM is more suitable for LTBI compared to RM. However, their limited availability and stringent ethical requirements further constrain the application of this model. These models can be used for preclinical pharmacological evaluation of novel anti-TB therapeutics and vaccines, particularly in assessing their efficacy in preventing LTBI reactivation. Current applications predominantly focus on investigating immunological alterations in TB-human immunodeficiency virus (TB-HIV) co-infected individuals and elucidating HIV-driven LTBI reactivation mechanisms, providing an important basis for predicting reactivation risk and timely anti-TB treatment in TB-HIV co-infected populations [88,89,90,91,92].

Current diagnostic criteria for LTBI models primarily rely on organ morphological changes, CFU counts in organ homogenates, and histopathological alterations assessed through Ziehl-Neelsen acid-fast staining (direct visualization of MTB bacillary load in tissues), hematoxylin and eosin (H&E) staining (evaluation of granuloma number and structural changes in organs), and immunohistochemistry analysis (detection of MTB antigens or associated cytokines) to establish the latent infection status. With the cross-disciplinary integration and development, magnetic resonance imaging (MRI) and positron emission tomography (PET/CT) have been used to monitor the disease progression of TB, which has greatly promoted the research on the expensive NHP LTBI model [93,94]. Due to variations in host susceptibility, MTB infection manifests differently across diverse populations (e.g., children, the elderly, and immunocompromised individuals). Consequently, a more comprehensive understanding of MTB and the mechanisms underlying TB reactivation is imperative. Standardized animal models and accurate, standardized evaluation methods play a pivotal role in this research.

## 3. Application of Animal Models in the Evaluation of TB Vaccine Efficacy

Animal models are a core tool in vaccine evaluation and development, with over 35 species of animals having been used in more than 1300 vaccine trials [95]. However, the selection of animal models still faces multiple challenges in the development of TB vaccines. Due to the widespread distribution of MTB in the host and the complex and diverse clinical phenotypes, there is currently a lack of standardized evaluation metrics that comprehensively reflect the pathophysiological characteristics of TB infection, which directly hinders vaccine development. Different types of TB (e.g., tuberculous pleuritis, tuberculous meningitis, and tuberculous peritonitis) require the selection of appropriate animal models based on their pathogenesis and the characteristics of the vaccine [96,97,98]. Animals exhibit varying sensitivities to challenge strains, and the virulence of the strains depends not only on the strain itself but also on the interaction with the host. Therefore, when selecting evaluation models, different challenge strains and animal types should be chosen according to specific needs. Due to the lack of effective model screening and evaluation methods, differences in challenge strains and animal types across models may lead to significantly varied results.

### 3.1. Rodents

Mice are widely employed not only in TB vaccine research but also in studies of MTB genomics, transcriptomics, and proteomics due to their low cost, rapid reproduction, long survival time, mature immune evaluation metrics, and the availability of commercial reagents as the most commonly used animal model in TB vaccine evaluation. For example, using mouse models to assess the virulence of specific MTB strains can provide valuable information on the genomic and epidemiological data related to TB outbreaks [99]. Challenging mice with different MTB strains can also link genomic differences and virulence factors between strains, enabling the exploration of MTB pathogenic mechanisms and virulence factors of highly virulent strains through bioinformatics analysis [100,101]. Currently, the most commonly used mouse strains include C57BL/6, BALB/C, C3HeB/FeJ, and DBA/2 [102].

C57BL/6 mice are used to test the immunogenicity and protective immunity of vaccine candidates during the early stages of vaccine development and closely resemble human TB clinical manifestations in terms of the distribution and immune responses in the liver and spleen. Their alveolar tissues contain a large number of macrophages and lymphocytes, and the synthesis and secretion of alveolar surfactants are enriched in lung tissues [103]. Additionally, C57BL/6 mice exhibit a high degree of macrophage and lymphocyte infiltration in the liver, making them an ideal animal model for studying the immune response mechanisms of MTB, vaccine-induced immune responses, and the pathogenesis of TB. The C57BL/6 mouse model holds great promise for research on the mechanisms of MTB infection, evaluation of therapeutic drugs, and assessment of vaccines.

Furthermore, this model holds substantial potential for evaluating TB vaccine efficacy, elucidating infection mechanisms, and screening therapeutic agents. However, their utility in screening vaccine immunogenicity is constrained by the limited diversity of expressed MHC molecules compared to humans. Specifically, C57BL/6 mice predominantly express MHC class II molecules, and certain MHC variants fail to present essential MTB antigens in a manner that elicits effective T-cell responses [104]. Consequently, subunit vaccines may fail to elicit immune responses in this model due to the absence of epitopes compatible with C57BL/6 MHC molecules. PPE15 only enhances the protective efficacy of BCG in C57BL/6 mice but has no effect in BALB/c mice demonstrates that differences also exist in protective efficacy evaluations [105]. The observation that PPE15 enhances the protective efficacy of BCG in C57BL/6 mice but not in BALB/c mice, demonstrating that differences exist in protective efficacy evaluations across different mouse strains. Therefore, initial screening of multiple mouse strains to identify candidates with potential immunogenicity, followed by selection of the F1 generation exhibiting optimal immunogenicity for vaccine efficacy evaluation, may represent a viable solution. The genomic heterozygosity of F1 mice confers distinct advantages: it mitigates interference from extreme immune phenotypes commonly observed in inbred strains while more closely mirroring the genetic diversity characteristics of the human MHC system (e.g., CB6F1). Although BCG provides similar protective effects in both BALB/c and C57BL/6 mice, it induces stronger Th1 and Th17 responses in C57BL/6 mice compared to BALB/c mice [106,107]. The susceptibility of BALB/c mice to H37Rv infection varies depending on the route of challenge, with common routes including intravenous injection, intraperitoneal injection, and aerosol infection. The commonly used doses were as follows: high dose, 1 × 10^6^–5 × 10^6^ CFU; medium dose, 1 × 10⁵–5 × 10⁵ CFU; low dose, 25–100 CFU; and ultra-low dose, 1–3 CFU [18,20,23,108]. Commonly used challenge doses for mice are not low, partly due to the inherent resistance of strains such as C57BL/6 to MTB. Human TB is thought to be elicited by an infectious inoculum of only 1–3 CFU. Studies indicate that ultra-low-dose challenge can assess vaccine-induced prevention of infection, enhance the discriminatory potential for differentiating vaccine protection levels, and provide immunity parameters potentially relevant to the clinical setting—parameters that cannot be evaluated using supra-physiological challenge doses [109,110,111]. Ultra-low doses better mimic the natural human infection state and facilitate the observation of vaccine protection. The challenge dose of < 10 CFU is commonly used to evaluate TB vaccines in NHPs. Consequently, the ultra-low-dose MTB infection model offers an improved platform for TB vaccine testing.

Different animal models, challenge methods, and doses may influence experimental outcomes. Therefore, the most reasonable experimental design should be selected based on the research objectives, the rationality of the challenge route, and the distribution of the strain in the target organs of the animals. Since different animal models may elicit varying immune responses, there is an urgent need to establish standardized animal models to make research results more reliable and applicable. Currently, TB vaccines in clinical trials such as AEC/BC02, M72/AS01E, and GamTBVac use C57BL/6 mice as their preclinical animal evaluation models. The protective efficacy of these vaccines is assessed using the standard H37Rv strain through aerosol infection and intraperitoneal injection, with challenge doses of 20–100 CFU and 1 × 10^5^ CFU, respectively (Table 1).

The C3HeB/FeJ mouse strain, a susceptible inbred model, is the first murine system to exhibit pulmonary pathology resembling human TB, including caseous granulomas and occasional cavities [112]. Human TB granulomas exhibit a heterogeneous cellular infiltrate comprising macrophages, T lymphocytes, and multinucleated giant cells, whereas C3HeB/FeJ mouse granulomas are predominantly composed of neutrophils and macrophages, with minimal T lymphocyte infiltration. Through classical breeding between susceptible and resistant strains, researchers developed congenic mouse lines and identified genetic loci linked to TB susceptibility and discovered that the sst1 locus encodes the transcription factor SP140, which enhances host resistance by suppressing type I interferon (IFN) responses [113]. Introgression of the sst1 susceptibility allele from C3HeB/FeJ into C57BL/6 mice results in necrotic granulomatous lesions resembling human pathology following low-dose MTB infection, enabling evaluation of genetic contributions to necrotic pathology [114]. In recent years, the C3HeB/FeJ model has been utilized to test drug efficacy and drug combinations, leveraging its human-like caseous necrosis to bridge preclinical and clinical outcomes, particularly in studying lesion pathology and drug interactions [115,116,117].

CBA/J mice, a substrain of the CBA lineage, exhibit heightened susceptibility to respiratory infections. Compared to the C57BL/6 mice, CBA/J mice are more prone to MTB infection and progression to active TB. This model has facilitated analysis of neutrophil dynamics in TB pathogenesis [118]. The susceptibility of CBA/J mice to MTB correlates with diminished interferon-gamma (IFN-γ) responses to immunodominant antigens such as Ag85 and early secreted antigenic target-6 (ESAT-6) [119].

Novel humanized mouse models recapitulate key features of human pulmonary tuberculous granulomatous responses, including granuloma formation, caseous necrosis, and bronchial obstruction following MTB infection, positioning them as valuable preclinical tools for evaluating anti-TB drugs and vaccines [120]. Humanized mice can better understand the infection process and characterize the human immune system response, commonly used to determine the immune response of vaccines to TMB infection [121]. Humanized mice are particularly effective for characterizing vaccine-induced immune responses to MTB infection, with both human CD4^+^ and CD8^+^ T cells demonstrating functional activation [122]. As a critical preclinical platform for assessing human T-cell immunity in TB vaccine development, humanized mice support the evaluation of diverse antigens and vaccine formulations, including hepatitis B vaccines, tetanus toxoid, and dinitrophenyl conjugates, which can elicit innate or adaptive immune responses [123,124]. Studies show that BCG vaccination combined with CpG-C molecular adjuvants in humanized mice induces distinct cytokines compared to those observed in C57BL/6 mice or guinea pigs, highlighting unique T-cell activation patterns [122]. Humanized mouse models also facilitate the study of HIV/MTB co-infection (e.g., NSG and NOG). Guohua Yi et al. [125] developed a novel humanized NSG-SGM3 mouse model for HIV/MTB co-infection, which recapitulates CD4^+^ T cell depletion, HIV viral load dynamics, and metabolic perturbations mirroring human co-infection patterns. Despite these advantages, humanized models face limitations, including xenogeneic graft-versus-host reactions that impair human T-cell survival, functional anomalies, and uncontrolled bacterial proliferation [126]. Furthermore, their utility is constrained by technical complexities, high costs, and ethical controversies surrounding human cell engraftment.

### 3.2. Guinea Pig

Guinea pig models recapitulate key features of human pulmonary pathology. Due to their high susceptibility to MTB, guinea pigs are widely employed in MTB infection studies, lymphocyte proliferation assays, skin reactivity assessments, and the evaluation of TB vaccine candidates [127,128]. Guinea pigs develop human-like granulomas post-MTB infection, characterized by progressive necrosis and lung damage analogous to human TB pathology [129,130]. Langerhans giant cells formed by macrophage and epithelioid cell aggregation have been observed in guinea pigs following mycobacterial infection, demonstrating their relevance for studying TB pathogenesis, primary/secondary lung injury, and therapeutic or vaccine interventions [61]. While some vaccine candidates underperform in mouse models, they demonstrate robust protection in both guinea pigs and humans. Typical challenge doses in guinea pigs (500–5 × 10^3^ CFU) are lower than those used in mice, enabling sensitive evaluation of TB vaccine efficacy. However, it remains higher than the physiological dose of normal infection. Currently, there are limited reports on guinea pig models with ultra-low challenge doses; the development of such models is essential for the stable and accurate evaluation of vaccines and the investigation of strain pathogenic mechanisms.

These models are now pivotal for evaluating TB severity and progression, informing therapeutic and preventive strategies [2]. However, their broader application is limited by the scarcity of species-specific immunological reagents, high experimental costs, and infrastructure demands. Additionally, species-specific immunoassay reagents such as cloned guinea pig cDNAs, IL-4 [131], IFN-γ [132], IL-10 [133], IL-17A [134], IL-1β, and MCP-1 [135] have been developed, enabling mechanistic exploration of host-pathogen interactions.

### 3.3. Rabbit

The rabbit model serves as an advantageous animal model for TB research due to its capacity to replicate multiple critical stages of human disease progression. Following MTB infection, pulmonary granuloma formation in rabbits closely mirrors human pathology, with a propensity for liquefaction and cavitation development [136]. Notably, rabbits exhibit lower susceptibility to H37Rv and Erdman infection compared to guinea pigs while demonstrating heightened sensitivity to *M. bovis*. This model has been extensively employed in vaccinology research, facilitating both the screening and evaluation of vaccine candidates, including BCG and subunit formulations. For example, it has enabled mechanistic investigations into H37Rv-induced cavitation pathogenesis, pathological progression, extrapulmonary dissemination patterns, and therapeutic interventions [137,138,139,140]. Researchers have confirmed the preclinical safety of VPM1002 by constructing a rabbit model to simulate the response of infants and young children to VPM1002 [141]. Comparative efficacy studies utilizing this model demonstrated superior protective outcomes with the TB subunit vaccine LT70 relative to conventional vaccine platforms [138,142].

The rabbit model demonstrates significant utility in research on spinal TB and TB meningitis. Its lumbar anatomical structure exhibits remarkable similarity to humans in both lesion manifestation and structural stability, rendering it widely adopted for therapeutic investigations such as evaluating doxycycline efficacy against TB spondylitis [143]. Consequently, the rabbit spinal TB model is recognized as the optimal platform for assessing anti-osteotubercular drug efficacy [144]. In tuberculous meningitis studies, this model effectively facilitates virulence assessment of MTB, disease progression analysis, and evaluation of novel vaccine candidates, particularly in therapeutic investigations involving first-line anti-TB regimens. Compared to vaccine evaluation, the rabbit model shows greater applicability for anti-TB drug research, enabling systematic exploration of pharmacokinetic parameters including drug penetration, tissue distribution, cellular accumulation within pulmonary lesions, and underlying pharmacodynamic mechanisms [145].

Although extensively employed in anti-TB drug research, rabbit models remain underutilized in preclinical vaccine development, with current clinical-stage TB vaccine candidates rarely employing this system. This limitation primarily stems from interspecies variations in MTB pathogenesis and virulence between rabbits and humans, compounded by rabbits’ relatively low MTB susceptibility [145]. Furthermore, technical challenges persist, including scarcity of immunological reagents and elevated maintenance costs. Fortunately, emerging applications of genomics and proteomics are expected to facilitate the development of susceptibility-enhanced rabbit TB models, potentially overcoming current limitations while reducing temporal and financial expenditures in TB research.

### 3.4. NHPs

NHPs demonstrate exceptional translational value in TB research due to their phylogenetic proximity to humans and heightened susceptibility to MTB. Following infection, NHPs recapitulate hallmark human pathological features, including caseous necrosis, liquefaction, and cavitation, alongside closely aligned immune responses [146,147,148]. The most widely utilized NHP models are rhesus macaques (RM) and cynomolgus macaques (CM). Walsh et al. [149] reported that CM exhibits stronger resistance to human-derived virulent MTB strains compared to RM. Langermans et al. [71] further demonstrated that BCG confers more robust protection against MTB infection in CM than in RM. Comparative studies revealed that RM develops more severe pulmonary and extrapulmonary lesions, higher bacterial loads, and accelerated disease progression post-MTB infection relative to Chinese CM [150]. Additionally, transcriptomic analyses of monocyte- and lymphocyte-associated genes have uncovered species-specific immunological divergences between RM and CM, providing critical insights into host-pathogen interactions within susceptible populations [151].

In recent years, NHP models have gained prominence in preclinical vaccine evaluation, particularly for assessing safety, immunogenicity, and protective efficacy. Common challenge routes include intratracheal instillation and aerosol infection. For instance, RM challenged via intratracheal instillation with 500 CFU of the Erdman strain were employed to validate the safety and protective efficacy of the M72/AS01E vaccine [42]. Preclinical evaluations of ID93:GLA-SE and MVA85A vaccines utilized Erdman strain challenges with low-dose (40–60 CFU) and high-dose (500–1 × 10^3^ CFU) regimens (Table 1). Low-dose aerosol infection models are increasingly adopted to better mimic natural human TB transmission dynamics and evaluate vaccine-induced immune responses. The use of high-dose challenge in non-human primate models may fail to demonstrate significant efficacy for vaccines that protect against natural low-dose infection, thus potentially overlooking their true protective potential. Stable and reproducible aerosol infection models using ultra-low doses (<10 CFU) of MTB have been established in both RM and CM. These models facilitate the development of precise MTB infection systems for evaluating novel TB vaccines, thereby enhancing clinical relevance and improving animal welfare [74]. These standardized protocols enable systematic comparisons of vaccine candidates while refining correlates of protection in complex host-pathogen systems.

NHP models further facilitate the testing of novel vaccine adjuvants. H56 fusion protein vaccine demonstrated the importance of adjuvant selection in TB subunit vaccines when evaluated as a BCG booster in NHPs [152]. Ultra-low-dose (8–16 CFU) aerosol challenges with the Erdman strain in macaques induced robust T-cell responses, but rAd5-boosted BCG regimens failed to enhance TB protection in rhesus macaques [153]. However, there is no clear standardized protocol for the development and research of TB vaccines in NHPs, and key challenges include optimizing NHP models, substituting with smaller animal models where feasible, and accelerating clinical translation without compromising safety or efficacy assessments. Addressing these gaps is imperative to refine preclinical-to-clinical pipelines for next-generation TB vaccines.

### 3.5. Ruminant

Bovines are one of the main hosts of MTB and develop granulomatous lesions and immune responses analogous to humans, coupled with slow clinical disease progression. Cattle can collect large amounts of blood after vaccination and challenge, and easily accessible immune reagents provide a solid foundation for cattle to evaluate the safety and efficacy of TB vaccines, explore the pathogenesis of TB, and develop novel vaccines [154]. *M. bovis* serves as the primary etiological agent of bovine TB, thus, bovine models are predominantly employed to study *M. bovis* pathogenesis and improve BCG vaccines. Additionally, cattle play a pivotal role in quantifying BCG efficacy, optimizing immunization protocols, and informing interventions to mitigate zoonotic TB transmission [155,156,157].

Bovine models are infrequently employed for non-*M. bovis* strains or non-bovine TB vaccine research due to high operational costs and the absence of cavitary lesion formation during infection. To address bovine TB epidemiology, novel infection models incorporating cattle and badgers have been developed because of the correlation between *M. bovis* infection in badgers and cattle [158,159,160]. Notably, bovine models have yielded clinically relevant insights in select vaccine evaluations; ID83 was identified as an immunological target in *M. bovis*-infected and BCG-vaccinated cattle, establishing a rationale for its inclusion in bovine subunit vaccines [161]. The modified vaccinia virus Ankara expressing antigen 85A (MVA85A), designed to boost BCG-induced immunity, demonstrated enhanced TB protection in calves through viral-vectored prime-boost regimens [25].

Goats emerge as a physiologically relevant model for TB research due to their susceptibility to *M. bovis* and *M. caprae,* with immune responses and pathological features closely mirroring human disease progression [64,162,163]. Infected goats develop caseous granulomas characterized by fibrosis, liquefaction, and cavitation. Goats of natural infection are almost similar to disease characteristics observed in both cattle and humans. Compared to cattle, goats offer distinct advantages, including a compact size, lower procurement costs, and reduced maintenance requirements. Figl et al. [164] evaluated the safety and immunogenicity of VPM1002 and its derivatives (PDX and NUOG) in juvenile goat models, demonstrating comparable safety to BCG and satisfactory immunogenicity. Vaccination routes such as subcutaneous, intramuscular, and intranasal administration have been systematically investigated, with evidence suggesting that differences in vaccination routes may affect the immune response characteristics of goats when evaluating the safety of TB vaccines. Challenge routes commonly employ aerosol, intratracheal, or endobronchial inoculation using *M. bovis* or *M. caprae*, with recommended dosing: low (1300–1500 CFU), medium (4.7 × 10^3^ CFU), and high (7.3 × 10^4^ CFU) [163,165,166]. Roy et al. [167] evaluated the immunogenicity and vaccine efficacy of BCG and MTBVAC via post-challenge of vaccinated goats exposed to *M. caprae*. While both vaccines reduced the severity of MTB-induced lesions, all vaccinated animals developed TB lesions at the end of the study.

### 3.6. Other Models

The zebrafish model has emerged as a valuable tool in TB research due to its susceptibility to *Mycobacterium marinum* (*M. marinum*), a non-tuberculous mycobacterium (NTM) that naturally infects poikilothermic animals such as fish and amphibians, causing systemic TB-like disease [64,168]. Zebrafish infected with *M. marinum* recapitulates key aspects of human TB and form necrotic granulomas histologically similar to human MTB granulomas. Both adult and larval zebrafish develop granulomas characterized by caseous necrotic cores surrounded by leukocytes and epithelial cells, with innate and adaptive immune response mechanisms to mycobacterial infection mirroring those observed in humans [169,170,171,172]. Zebrafish serves as a surrogate vertebrate system for vaccine development [173]. For instance, a DNA vaccine combining Ag85, ESAT-6, and CFP-10 antigens demonstrated robust protection in zebrafish, consistent with efficacy observed in other animal models [174].

Zebrafish embryos are uniquely suited for high-throughput anti-TB drug screening, enabling early-stage evaluation of novel compounds for in vivo toxicity and therapeutic efficacy [175,176,177]. A recent review by Antunes et al. [178] comprehensively outlined the utility of zebrafish models in accelerating anti-TB drug discovery. Furthermore, zebrafish have proven instrumental in elucidating mycobacterial pathogenesis, particularly through *M. marinum* infection studies investigating mechanisms underlying extrapulmonary TB manifestations such as tuberculous meningitis and ocular TB, as well as identifying virulence-associated genetic determinants. Zebrafish models are typically challenged via intraperitoneal injection with *M. marinum*. and common challenge doses include low-dose (20–30 CFU), high-dose (1 × 10^4^ CFU), and lethal-dose (2 × 10^4^–2.7 × 10^4^ CFU) regimens [170,174]. However, the absence of universally accepted infection protocols and dose standardization limits cross-study comparability. A critical constraint arises from the predominant use of *M. marinum* rather than MTB in zebrafish models, and there are few reports of MTB being used in zebrafish models. This species-specific limitation underscores the need for standardized methodologies to enhance translational relevance across different animal models and bacterial strains.

Parikka et al. [179] established the first zebrafish LTBI model and induced latent infection in adult wild-type zebrafish via intraperitoneal injection of 35 CFU *M. marinum,* and the model was activated by dexamethasone-induced immunosuppression four weeks post-infection. Although zebrafish LTBI models remain understudied, they demonstrate significant potential for LTBI modeling due to their low establishment cost, rapid experimental timelines, and easy genetic manipulation that collectively facilitate mechanistic investigations of mycobacterial persistence and reactivation. The utilization of *M. marinum* as a surrogate for MTB eliminates biosafety containment requirements, thereby significantly reducing operational costs and infrastructure demands [180]. Zebrafish embryos enable real-time visualization of host immune responses to *M. marinum* infection through fluorescent labeling of immune cells and fluorescence-tagged *M. marinum* strains to screen antibacterial activity drugs by quantifying bacterial load [181,182]. However, primarily employed for studying innate immunity, it fails to recapitulate adaptive immune responses critical to human TB pathogenesis; the absence of pulmonary and lymphoid tissues fundamentally restricts its capacity to model human disease progression. Most critically, the suitable survival temperature for *M. marinum* is 28 –32 °C and it is difficult to survive above 37 °C. This means that its growth will be inhibited in the human body and other isothermal animals. This difference raises concerns about extrapolating vaccine efficacy data from zebrafish to humans.

In recent years, *Galleria mellonella* (GM) has emerged as an invertebrate model in TB research, finding application in fundamental mechanistic studies and drug screening due to its unique advantages. The GM larvae system serves as a reproducible, cost-effective, high-throughput, and ethically acceptable infection model that circumvents limitations of traditional mammalian models [183]. Unlike zebrafish or Drosophila, GM can be incubated at 37 °C without specialized equipment. Quantitative inoculation with bacterial suspensions enables observation of dose-dependent larval mortality and changes in bacterial load, allowing quantitative assessment of strain-specific pathogenicity [183]. This model has been employed to infect with bioluminescent *M. bovis* BCG for evaluating drug efficacy [184]. The GM model plays an important role as an alternative host for studying mycobacterial infection pathogenesis and elucidating host-mycobacterium interactions, including latent TB infection [185]. It provides a pre-screening platform for assessing drug efficacy/toxicity and determining mycobacterial virulence prior to using conventional mammalian models. Studies have found that infecting GM with H37Rv, BCG, and clinical isolates (e.g., SAMTB) at doses of 1 × 10^6^–1 × 10^7^ CFU demonstrates distinguishable virulence profiles based on survival kinetics and bacterial loads within 96–192 h [186]. GM offers broad potential for studying MTB infections under BSL-2 conditions, eliminating constraints of BSL-3 research [187]. Although GM exhibits shorter latency periods and requires higher infection doses than mammalian models, its rapid lifecycle, low cost, minimal maintenance, and experimental speed confer significant advantages for preliminary investigations.

However, as an emerging animal model, GM faces limitations due to the scarcity of widely available immunological and molecular reagents, hindering comprehensive characterization of host responses to infection. Its inability to recapitulate T-cell-mediated immunity (e.g., granuloma maturation) restricts its utility for studying latent TB infection or reactivation mechanisms. Furthermore, GM fails to replicate hallmark human TB pathologies such as caseous necrosis or cavitation and only reflects the acute infection phases. Collectively, GM serves as a complementary tool rather than a replacement model in TB research. It remains suitable for preliminary drug toxicity/efficacy screening, rapid virulence gene identification, and innate immunity investigations. Future advancements may combine transcriptomics with high-sensitivity bioluminescent imaging to elucidate mechanisms of novel TB vaccines and drugs [184]. Experimental outcomes exhibit sensitivity to larval sourcing, weight variations, and injection techniques, necessitating stringent standardization protocols to ensure reproducibility. Establishing standardized operating procedures (e.g., larval strains, infection doses) is critical to enhance result reproducibility and cross-study comparability.

## 4. Standardization of Protective Efficacy Evaluation Models

### 4.1. Standardization of Preclinical Models

**Figure 2:**
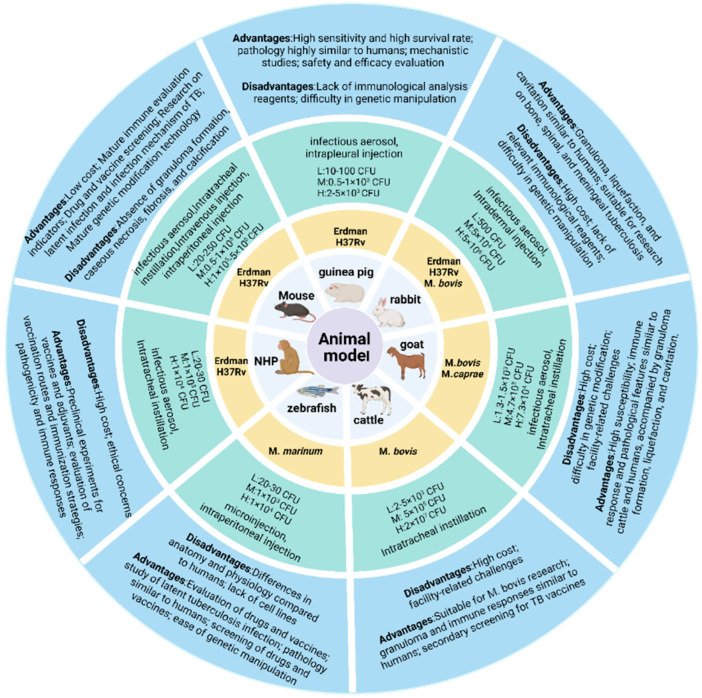
Animal challenge models. The models involve the commonly used challenge strains, infection routes, and low/medium/high dose ranges for each animal model, along with their respective advantages and limitations.

While numerous animal models exist for MTB research, standardized protocols remain elusive, with significant geographical and methodological variations persisting across studies. The inherent biological specificity of animal models necessitates context-specific selection criteria to ensure experimental validity. For established models, rational selection based on experimental objectives is critical to generate reliable data. As illustrated in Figure 2, we summarize current evaluation metrics for common infection models, among which mice and guinea pigs are the most commonly used TB vaccines and drug evaluation models and have always been an important component of animal models. Mice are predominantly utilized for preclinical screening of TB vaccine candidates and evaluation of protective efficacy. While post-MTB infection immune responses partially recapitulate human immunobiology, these models exhibit limitations in modeling disease progression and pathological lesion development. Female mice aged 6–8 weeks with controlled body weights (16–20 g; intra-group variation ≤ 2 g) are recommended. Guinea pigs are routinely employed to assess cutaneous reactivity (e.g., MTB antigen-based skin test (TBST), tuberculin skin test (TST), and IGRA), evaluate the protective efficacy of TB vaccine candidates, and investigate transmission dynamics of drug-resistant MTB [188,189,190,191]. In challenge experiments, guinea pigs exhibit greater inter-individual variability compared to mice; therefore, cohort sizes should be determined based on statistical requirements. Standardized housing conditions may mitigate experimental variance.

Rabbit and nonhuman primate (NHP) models develop granulomas, liquefaction, and cavitation following MTB infection, serving as critical platforms for vaccine candidate screening and evaluation. Rabbit models are primarily employed to assess BCG and subunit vaccine efficacy, as well as to investigate mechanisms of cavitation formation. NHPs, such as rhesus macaques, are utilized for preclinical evaluation of TB vaccine safety, immunogenicity, and protective efficacy. However, rabbits and NHPs are more difficult to meet the statistical requirements for animal numbers compared to small animals such as mice. The primary condition for standardizing animal models is to select suitable animals, maintain consistency in animal specifications and status as much as possible, and ensure reproducibility and cross-laboratory comparability of the experimental process and results in order to provide a reliable basis for scientific research and clinical translation.

### 4.2. Challenge Strains

The selection of standardized challenge strains for assessing TB vaccine efficacy remains limited (Figure 2). It is recommended that standardized infection strains be obtained from institutions such as the American Type Culture Collection (ATCC), Biodefense and Emerging Infections Research (BEI) Resources Repository, and the China Medical Culture Collection (CMCC). After prolonged in vitro culture, the replicative capacity of clinical strains in animal models decreases [192,193]. The culture medium may be unsuitable for long-term maintenance of strain virulence, necessitating timely reactivation of the strains. All strains used in animal models should be sequenced before use, as each commonly used MTB strain has numerous substrains, and significant differences in genetic sequences can exist among the same strain from different sources. Additionally, their abilities to grow to high levels and virulence vary, which can lead to differences in research outcomes. More importantly, the growth method of the strain can also affect its ability to function optimally in animal models. Strains cultured using liquid medium (7H9) and solid media (7H10, 7H11, and Löwenstein-Jensen medium) may exhibit different morphological and biological characteristics due to the distinct environments of in vitro culture and in vivo challenge in animals. The bacterial suspension prepared from strains obtained through solid culture is not a completely single-cell suspension, which can affect the accuracy of the challenge dose. In addition, long-term preservation of strains can reduce cell viability. Cooling rate and storage temperature are the most critical factors affecting cell viability, while preservation using different culture media has no significant effect on the strains [194]. Therefore, long-term preserved strains should be re-counted for CFU before use.

When comparing different strains, it is necessary to standardize whether the strains have been reinforced in animals and confirm whether their phenotypes are stable. However, relying solely on a single MTB strain for vaccine and drug evaluations compromises the generalizability of results, as clinical isolates exhibit substantial genetic and phenotypic diversity. Geographic variations in circulating strains (e.g., Beijing vs. Euro-American lineages) may lead to differential vaccine and drug responses across populations. Furthermore, the acquisition of drug resistance is frequently accompanied by concurrent changes in bacterial virulence and fitness. Different drug-resistant strains often display distinct virulence gene expression profiles and pathogenic potential, resulting in varied clinical manifestations post-infection [195]. Compared to fluoroquinolone-sensitive multidrug-resistant TB (MDR-TB), extensively drug-resistant TB (XDR-TB) is associated with a higher frequency of cavitary lung lesions and longer treatment duration [196]. Badamasi et al. [197] identified a significant association between IL-8 genotypes and both neurotoxicity susceptibility and pulmonary TB disease risk [198]. Patients infected with the Beijing strain more frequently develop fever independent of drug resistance, virulence, or disease severity, while demonstrating enhanced tolerance to anti-TB drugs. These findings suggest that the Beijing strain may possess distinct pathogenic properties [199,200,201]. Beijing strains (Lineage 2) demonstrate sustained intracellular survival and unimpaired growth within macrophages, whereas other phylogenetic lineages exhibit restricted proliferation under these conditions [202]. Notably, studies have shown that a potential epidemiological association between Beijing strains and TB co-infection in people living with HIV (PLWH) [203]. Highly prevalent Beijing strains exhibit enhanced virulence compared to low-prevalence variants, with distinct immunomodulatory profiles: high-virulence strains induce strong but transient TNF-α expression during early infection, followed by elevated IL-4 production in later stages; low-virulence strains demonstrate progressive IFN-γ and TNF-α expression throughout infection [204]. Olivier et al. [205] demonstrated that the Beijing strain induces significantly lower levels of IL-6, TNF-α, IL-10, and GRO-α compared to H37Rv. Furthermore, prophylactic BCG vaccination demonstrated inferior protective efficacy against Lineage 2 isolates compared to Lineage 4 isolates in a C57BL/6 mouse model challenged intravenously [206]. Researchers systematically evaluated virulence heterogeneity among MTB (H37Rv, SA161, HN878, CDC1551, and Erdman) using a C57BL/6 mouse model of whole-body aerosol infection, revealing different virulence factors and drug resistance between different lineages [207,208].

The Beijing genotype strains demonstrate a heightened case clustering tendency and remain the predominant circulating strain in China [209]. However, there is currently a critical lack of standardized Beijing genotype strains suitable for evaluating the protective efficacy of TB vaccines in preclinical models, despite their epidemiological dominance and distinct pathobiological characteristics in high-burden regions. Strain diversity plays a pivotal role in advancing multi-epitope TB vaccine platforms and enhancing the reliability of preclinical vaccine evaluations. To address the critical limitation of homogeneous strain utilization in current practices, there is an urgent need to establish a standardized reference strain library that assesses genetic diversity through whole-genome sequencing of epidemiologically relevant strains. Systematically evaluates the efficacy of vaccine candidates against phylogenetically distinct lineages. Reference strains spanning diverse drug resistance profiles (e.g., MDR/XDR) and major phylogenetic lineages (Lineage 2 Beijing, Lineage 4 Euro-American).

### 4.3. Challenge Routes and Doses

The evaluation of TB vaccine protective efficacy must account for variations in challenge routes and doses, as different challenge methods and dosing regimens significantly influence host susceptibility and vaccine-mediated protection. We have summarized the infection methods and challenge doses across various animal models from existing studies (Figure 2). It is worth noting that the accuracy of the challenge dose significantly affects the evaluation results, especially when comparing the protective efficacy of different vaccines. Therefore, to ensure the accuracy of the results, it is necessary to verify the viable bacterial count in the inoculum, and it is preferable to filter the inoculum through a 5 μm membrane to obtain a single-cell bacterial suspension. In the case of aerosol infection, it is essential to ensure uniform particle size of the aerosol, typically ≤ 5 μm in diameter and common aerosol infection equipment can meet this standard [210]. To simulate natural MTB infection, aerosol challenge remains the ideal route. However, for larger animal models (e.g., NHPs, cattle), intratracheal instillation is often employed due to infrastructure constraints associated with biosafety and aerosol delivery systems. The route of infection critically determines pathological outcomes, with aerosol challenge inducing the most severe pulmonary pathology, while intraperitoneal or intradermal challenge results in predominant splenic involvement. Intravenous tail vein injection has been utilized to treat MTB infection in rodents (e.g., mice); however, this method faces technical challenges, including high difficulty of tail vein puncture, low throughput, and poor organ specificity. In contrast, aerosol challenge, intratracheal instillation, and intranasal inoculation enable lung-targeted infection with well-defined pulmonary pathology, making them preferable for modeling respiratory TB. Consequently, infection route selection must align with research objectives, for instance, distinct animal models and challenge methods may be required to study extrapulmonary manifestations such as TB meningitis or osteomyelitis [211,212,213].

Tsenova et al. [214] investigated the protective efficacy of BCG vaccination in rabbits challenged with varying degrees of the hypervirulent HN878 strain versus the hypovirulent CDC1551 strain. Their findings demonstrated that HN878 infection induced significantly higher pulmonary fibrosis lesions compared to CDC1551 and indicated that fibrosis may be a characteristic of HN878 strain infection in the host. Notably, BCG-immunized rabbits achieved localized containment of low-dose HN878 challenge but failed to confer protection against higher challenge doses [214]. These findings collectively demonstrate that strain-specific virulence traits and challenge dose thresholds directly impact vaccine-mediated protection, underscoring the necessity of incorporating hypervirulent strains in preclinical evaluation frameworks. The absence of universally standardized inoculum doses across MTB challenge models remains a significant barrier to experimental reproducibility in TB research. Harmonization of infection routes (e.g., aerosol, intravenous, intratracheal) and dose regimens is imperative to ensure cross-study comparability, particularly given the strain- and lineage-specific variations in organ tropism and pathogenicity. The absence of universally standardized challenge routes and doses across MTB challenge models remains a significant barrier to experimental reproducibility in TB research. Harmonization of infection routes (e.g., aerosol, intravenous, intratracheal) and dose regimens is imperative to ensure cross-study comparability, particularly given the strain-and lineage-specific variations in organ tropism and pathogenicity.

Through systematic review of established animal models across infection routes, we selected the acceptable challenge dose range by balancing the infection success rate and the survival period; the specific range of challenge doses can be referred to in Figure 2. For highly susceptible models such as guinea pigs and non-human primates, corresponding infection doses need to be explored and established according to the infection route (subcutaneous/intraperitoneal/nasal drop/aerosol, etc.). Generally, the dose needs to be reduced to 1/100–1/10 of the mouse equivalent, or even lower. Although ultra-low-dose models (<10² CFU) are increasingly studied, conventional doses remain predominant in preclinical vaccine evaluations due to the success rate being unstable when infected with ultra-low doses. It is expected that more stable ultra-low challenge dose models will be used in the evaluation of the protective efficacy of TB vaccine candidates in the future.

### 4.4. How to Evaluate

The key detection indicators for evaluating the protective efficacy of tuberculosis vaccines include infection efficacy monitoring, phenotypic monitoring, and pathological and immunophenotypic detection. Although different animal models exhibit variability, if a vaccine demonstrates good protective efficacy in different models, its stability can be proven. Infection efficacy monitoring is generally reflected by the infection success rate. Low-dose infection may lead to an excessively low infection success rate, while high-dose infection may cause animals to die too quickly. Appropriate infection doses can be explored independently, and animal survival time can be recorded if necessary. Phenotypic monitoring refers to the physical changes in animals during challenge, including body weight, body temperature, humidity, lighting, and feed intake changes. In addition, cage boxes and feed need to be replaced regularly, preferably 1–2 times per week. Pathological detection involves counting the number of granulomas in the lungs, liver, and spleen and calculating the bacterial load in organs through tissue homogenate culture. Immunophenotypic detection refers to detecting the proportion of CD4⁺/CD8⁺ T cell subsets and the differentiation status of memory T cells in animals by flow cytometry. Infection serum can be used to detect antigen-specific antibody concentrations (IgG/IgG1/IgG2a) and cytokines (IFN-γ, IL-2, TNF-α) [215,216].

Currently, the assessment of TB vaccine protective efficacy remains constrained by incomplete standardization of evaluation metrics, with variability in preclinical model selection (e.g., murine vs. nonhuman primate systems), single challenge strain characteristics (H37Rv), and route/dosage parameters (aerosol vs. intravenous) serving as primary determinants of outcome heterogeneity. Divergent pathological manifestations across species and host susceptibility thresholds to MTB infection limit cross-model comparability and undermine the translational relevance of preclinical findings. Therefore, researchers should establish a more comprehensive evaluation system, enhance in-depth research on clinical translation relevance, and conduct preclinical pharmacodynamic evaluation studies scientifically and rationally. By balancing the research efficiency of different animal models with clinical translation prediction, effective evidence across multiple species can be provided for vaccine development.

## 5. Conclusions

Vaccination remains the most cost-effective strategy for TB prevention and a critical pathway toward achieving the WHO’s 2035 End TB Strategy. However, despite over a century of use, no TB vaccine has surpassed the protective efficacy of the BCG vaccine. While certain TB vaccine candidates elicit robust protective responses in animal models, their clinical efficacy in humans has proven inconsistent. This translational gap stems from critical limitations in model standardization: high-TB-burden countries often lack infrastructure for advanced models (e.g., controlled aerosol challenge systems), restricting the use of larger animal models such as NHPs that better approximate human pathology. Furthermore, the absence of consensus guidelines specifying strain-matched animal models for distinct vaccine platforms has led to suboptimal model selection in preclinical studies. Currently, there is no clear regulation on which animal models need to be used for different types of TB vaccines, resulting in the unreasonable use of animal models in preclinical research. Therefore, standardization of animal models can help solve the current problems.

The selection of challenge strains—encompassing strain lineage, virulence profile, genetic homogeneity, phenotypic consistency, and in vivo stability—serves as the primary determinant of evaluation model fidelity, thereby critically influencing the validity of pharmacodynamic assessments in TB vaccine development. MTB exhibits marked geographical stratification in its global distribution, driven by environmental adaptations and host selection pressures, resulting in significant genotypic and phenotypic heterogeneity among circulating strains across diverse endemic regions. Although numerous animal models are currently available for MTB challenge research, the predominant reliance on a single standard strain represents a critical limitation in pharmacodynamic evaluations.

Consequently, there is an urgent need to systematically identify and characterize region-specific clinical isolates through comprehensive genomic sequencing (e.g., Illumina/Nanopore WGS), phenotypic profiling (e.g., drug susceptibility testing), and virulence assessment (murine LD50 determination). This process aims to establish a library of candidate strains exhibiting high and stable virulence, genetic stability, and complete genomic annotation for standardized preclinical evaluation of vaccine candidates. Distinct MTB genotypes exhibit differential organotropism and virulence patterns, manifesting as varied pathological manifestations across target organs. It is worth exploring how to establish a standardized system for animal models, strains, and experimental conditions to break through the bottleneck of TB vaccine development. In addition, the research and development difficulty of the TB vaccine lies in how to screen out effective antigens. The screening of antigens is closely related to animal models and challenge strains. In conclusion, the standardized research on animal models and strains may accelerate the screening of new antigens more quickly. It is also hoped that more standardized animal models and reference strains can be used in the research and development of vaccines and drugs, with the aim of successfully achieving the goal of ending TB by 2035.

## Figures and Tables

**Table 1:** Preclinical protective efficacy evaluation models. This table is sorted in alphabetical order and covers most of the current research on the protective efficacy of TB vaccines that have entered clinical trials. CMCC: National Center for Medical Culture Collections.

Vaccine	Phase	Animal Model	Challenge Strain	Challenge Route	Challenge Dose/CFU	References
AEC/BC02	Ⅱa	Guinea pig	CMCC 95052	Subcutaneous injection	100–1 × 10^3^, 5 × 10^3^	[16,17]
ChAdO×1.85A +MVA85A	Ⅱa	BALB/c	H37Rv	Aerosol challenge	250	[18]
		Guinea pig	H37Rv	Aerosol challenge	10–50, 500–1000	[19]
		BALB/c, Guinea pig, Rhesus monkey	H37Rv	Aerosol challenge	500	[20]
		BALB/c	H37Rv	Intravenous injection	2 × 10^5^	[21]
		Rhesus monkey	Erdman	Aerosol challenge	40–60	[22]
		Rhesus monkey	Erdman	Intratracheal instillation	1000	[23]
		BALB/c	H37Rv	Intraperitoneal injection	1 × 10^6^–5 × 10^6^	[24]
		Cattle	*Mycobacterium bovis* (*M. bovis*)	Intratracheal instillation	2 × 10^3^	[25]
DAR-901	Ⅱb	C57 BL/6	H37Rv	Aerosol challenge	50–100	[26]
GamTBVac	Ⅲ	C57BL/6, Guinea pig	H37Rv	Aerosol challenge	1 × 10^3^	[27]
H1: IC31/ H1: CAF01	Ⅰ	BALB/c×C57BL/6 (CB6F1)	H37Rv/ Erdman	Aerosol challenge	50	[28]
H4: IC31	Ⅱ	Guinea pig, CB6F1	Erdman	Aerosol challenge	10–50, 50	[28,29]
H56: IC31/H56: CAF01	Ⅱb	CB6F1	H37Rv	Aerosol challenge	50	[28]
H107e/CAF10b	Ⅰ	CB6F1	Erdman	Aerosol challenge	50–100	[30]
ID93+GLA-SE (QTP101Q)	Ⅱa	SWR/J, C57BL/6	H37Rv	Aerosol challenge	50–100	[31,32]
		Rhesus monkey	Erdman	Intratracheal instillation	500	[33]
		C57BL/6, Guinea pig	H37Rv, TN5904	Aerosol challenge	50–100, 20–50	[34]
		C57BL/6	Beijing MTB K	Aerosol challenge	150, 200	[35,36]
LT69/LT70	/	C57BL/6	H37Rv	Aerosol challenge	50–100	[37,38]
MTBVAC	Ⅲ	C57/BL6	H37Rv	Aerosol challenge	150	[39]
M72/AS01E	Ⅲb	C57BL/6	H37Rv	Aerosol challenge	20–50, 50–100	[40,41]
		C57BL/6, Rhesus monkey	Erdman	Intratracheal instillation	500	[42]
		Guinea pig, C57BL/6	H37Rv	Aerosol challenge	20–100	[43]
RUTI^®^	Ⅱb	C57BL/6, Guinea pig	H37Rv	Aerosol challenge	20–50/10	[44]
		DBA/2, C57BL/6	H37Rv	Aerosol challenge	20–50	[45]
		C57BL/6	H37Rv	Intraperitoneal injection	1 × 10^5^	[46]
		Guinea pig	H37Rv	Intrapleural injection	2 × 10^3^	[47]
TB/Flu-05E	Ⅰ	C57BL/6	H37Rv, Erdman	Subcutaneous injection	1 × 106	[48,49,50]
VPM1002	Ⅲ	BALB/c	H37Rv, Beijing MTB W	Aerosol challenge	100–200, 50–100	[51]

## Data Availability

Not applicable.
